# Feasibility of non-imaging, random-sampling second harmonic generation measurements to distinguish colon cancer

**DOI:** 10.1117/1.BIOS.1.3.035001

**Published:** 2024-11-26

**Authors:** Jenna Montague, Lucas Young, Hasina Shir, Travis Sawyer, Joshua Routh, Valentine Nfonsam, Jennifer K. Barton

**Affiliations:** a University of Arizona, James C. Wyant College of Optical Sciences, Tucson, Arizona, United States; b University of Arizona, Department of Biomedical Engineering, Tucson, Arizona, United States; c University of Arizona, Health Sciences, Tucson, Arizona, United States; d Midwestern University, Department of Pathology, Glendale, Arizona, United States; e University of Arizona, Department of Surgery, Tucson, Arizona, United States

**Keywords:** collagen, second harmonic generation, multiphoton microscopy, colorectal cancer, non-imaging, random sampling

## Abstract

**Significance:**

In the United States, colorectal cancer is the third leading cause of cancer death. Colonoscopy with polyp removal may suffer from incomplete resection. Collagen is altered in dysplastic tissue and can be studied with second harmonic generation (SHG) imaging. SHG imaging endoscopes require miniaturized scanning components, which greatly adds to endoscope complexity.

**Aim:**

We investigate whether non-imaging, randomly sampled SHG line or point intensity measurements are sufficient to distinguish normal tissue from tumor and tumor-adjacent tissue.

**Approach:**

Unstained tumor, normal, and tumor-adjacent thin sections from 10 colorectal cancer subjects were imaged using a multiphoton microscope with constant power. SHG signal from collagen was isolated by grayscale thresholding, and the grayscale mean of the image was calculated. Supra-threshold pixels and lines of pixels in the image were randomly selected to simulate point sampling and line scanning.

**Results:**

The mean SHG signal from normal samples was significantly greater than adjacent samples (p<0.05) and tumor samples (p<0.01). For both sampling types, the p-value becomes reliable after randomly sampling only 1000 times.

**Conclusions:**

Reliable cancer detection information may be obtained through non-imaging SHG intensity measurements. A simple endoscope with this capability could help identify suspicious masses or optimum surgical margins.

Statement of Discovery We report on a novel non-imaging second harmonic generation measurement technique that can differentiate colorectal tumors from normal colon tissue without a scanning system.

## 1 Introduction

A U.S. study on cancer incidence and mortality projected that colorectal cancer (CRC) is expected to be the third most diagnosed cancer in the country and the third leading cause of cancer death in 2024.^1^ The current standard diagnostic technique for colon cancer is a colonoscopy-guided biopsy.^2^ Although generally accurate for the detection of polyps, colonoscopy has a high rate of incomplete resection of neoplastic polyps (15.9% to 20.8%), which can result in post-colonoscopy colorectal cancer.^3^ This deficiency indicates a strong need for additional tumor margin verification techniques to determine polyp resection boundaries. Colonoscopes have a small working channel through which diagnostic tools can be easily implemented, which could decrease incomplete resection rates without adding undue complexity to the procedure.^4^

### 1.1 Collagen and Second Harmonic Generation

Tumor cells grow in an uncontrolled manner, in part because of biochemical and mechanical changes in their microenvironment, the extracellular matrix. Collagen, a major structural component in the extracellular matrix, has been shown to have a pivotal role in cancer development.^5^ Studies have shown that structural changes happen in the collagen surrounding a tumor that can be difficult to detect with traditional histology.^6^ Additional methods for studying the structural changes in collagen associated with cancer development can provide insight into cancer diagnosis and progression status.

Second harmonic generation (SHG) can be used to quantitatively study collagen’s structural changes. SHG is a multiphoton optical phenomenon that certain nonlinear materials, including collagen, can produce through a scattering mechanism.^7^^,^^8^ When two photons of the same frequency simultaneously interact within these materials, one photon with double the energy and half the wavelength of the original photons may be produced. The intensity of the SHG signal that collagen can produce is dependent on the nonlinear optical susceptibility of the collagen.^9^ Nonlinear optical susceptibility depends on the thickness of fiber thickness, fiber density, and fibril “packing,” which is the three-dimensional structure of the fibrils that make up collagen fibers. Many studies have used backscattered SHG and other backscattering techniques as quantitative indicators of extracellular matrix alterations in various cancer types, including CRC.^10^^–^^14^ Researchers have also shown that an SHG directionality comparison between the forward- and backward-scattered signal can be utilized *ex vivo* as an independent prognostic indicator for metastasis.^15^^,^^16^

There are two main advantages to studying collagen with SHG. The primary advantage is that it can serve as a label-free imaging technique that visually isolates collagen from its surroundings because collagen is the dominant material in tissue that produces a significant SHG signal.^7^^,^^8^ The secondary advantage is that to produce a strong SHG signal, it is necessary to confine the excitation light to a small focal volume to increase the probability of a two-photon effect taking place. Given this selective excitation only within this small focal volume, three-dimensional information about the collagen structure can be easily obtained by gathering intensity data from each focal volume and combining these individual voxels (volumetric pixels) to construct a three-dimensional image.^7^^,^^8^

### 1.2 Multiphoton Microscopy

The data for an SHG image is traditionally obtained *ex vivo* with a multiphoton microscope.^17^ The key components of a multiphoton microscope are a femtosecond laser, a high numerical aperture (NA) focusing lens or objective, and scanning components that move the laser beam, and thus, the focal volume, across the field of view. Lateral scanning usually is accomplished with galvanometer-based mirrors that rotate to move the beam across the field of view.^18^ Alternative scanning methods may maximize acquisition speed such as resonant galvanometer systems,^19^ hexagonal mirror scanning,^20^ and microelectromechanical systems (MEMS) mirrors.^21^

### 1.3 Multiphoton Endoscopy

Our group and others have previously obtained multiphoton images through an endoscopic architecture.^22^^,^^23^ Because scanning mirrors and MEMS devices are challenging to fit in the distal assembly of very small diameter endoscopes, we used a tube piezoelectric device with a cantilevered fiber. However, miniature two-dimensional scanning mechanisms are generally expensive and complex and may create a scan pattern that deviates from the standard rectilinear grid. There is therefore a need for a simpler way to obtain SHG intensity data endoscopically. This study investigates to what extent scanning is necessary to obtain meaningful SHG intensity data for the purpose of colorectal cancer diagnosis. We simulate random one-dimensional line-scanning and random point-sampling techniques (Table 1). As opposed to traditional two-dimensional synchronized scanning, these techniques can be implemented with one-dimensional synchronized scanning (e.g., spectro-temporal encoded methods^24^) or non-synchronized manual point sampling (e.g., endoscopist’s random hand movements), respectively. We hypothesize that non-imaging, randomly sampled SHG intensity measurements are sufficient for differentiating between tumor and normal tissue.

**Table 1:** Simulated image types, corresponding data localization type, and potential scanning hardware.

Description	Data localization	Hardware
Traditional imaging	Two-dimensional (image)	2D Galvos, MEMS, piezoelectric
Random line-scanning	One-dimensional (lines)	1D Galvo, spectral encoding
Random point-sampling	Random (points)	Manual agitation

## 2 Methods

### 2.1 Sample Preparation

Surgically resected colon tissue samples from 10 colon cancer subjects were obtained under a University of Arizona Institutional Review Board-approved protocol. Patients gave informed consent. Three different samples were obtained from each resection: tumor, tumor-adjacent, and normal. Tumor samples were obtained from the bulk of the tumor. Tumor-adjacent samples were taken from the first normal-appearing (to the physician) tissue adjacent to the tumor. Finally, normal tissue was obtained from the edge of the resection where the physician assumed a clear margin, typically 1 cm or more away from the tumor. The samples were fixed in 10% formalin, embedded in paraffin, cut into 6-μm thick sections using a microtome, and placed on glass slides. Serial dilutions of ethanol were used for deparaffinization, and the slides were stored in water until imaging.

Additional adjacent sections were stained with hematoxylin and eosin, and a diagnosis was provided by a pathologist. Pathology diagnosis of the actual imaged tissue sample could differ from the overall patient diagnosis.

### 2.2 Imaging

Images were obtained with a Zeiss LSM 880 multiphoton microscope with linearly polarized 850 nm excitation from a tunable Spectra-Physics Mai Tai DeepSee laser (tunable range 690 to 1040 nm). The objective was a 30X/0.8NA Plan-Apochromat (Zeiss #440640-9903-000), located in a backward (epireflectance) orientation. Emission from 400 to 430 nm was collected using a bandpass filter embedded in the microscope. The image size was 1024×1024  pixels over 425×425  μm. Power was kept constant. Because slides were only 6-μm thick and axial resolution is limited to about 1 to 2  μm, only one image plane was obtained, resulting in two-dimensional data.

One region per slide was randomly selected by the microscope user with the selection criteria being (1) the image contained mucosa only and (2) no image artifacts were present from sectioning. The mucosa is the most luminal layer of the colon and is thus considered to be comparable with the tissue that would be imaged by an endoscope *in vivo*.

### 2.3 Data Analysis

**Fig. 1:**
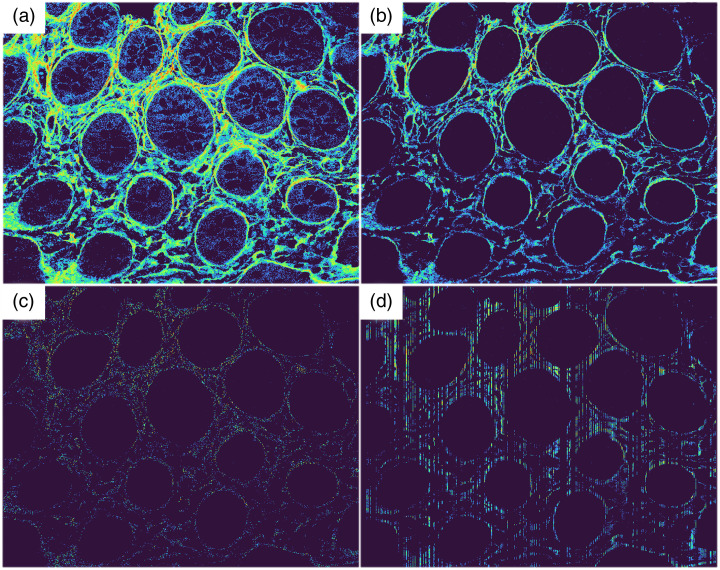
Representation of colon mucosa analysis algorithm with enhanced brightness and contrast. Starting with the raw image (a), the image was thresholded to exclude low- and high-value pixels (b), point-sampled (c), and line-sampled (d). Panel (c), 200,000 pixels, and panel (d), 400 lines, were chosen for easy visualization of the algorithm. Different sample sizes (10, 100, 1000, and 10,000) were implemented in actual data processing. Image size 425×425  μm.

Once images were obtained, the data from each image were processed in MATLAB. As shown in Fig. 1, low and high thresholds were applied that discarded pixel values that were <4 (background noise and autofluorescence leakage into the SHG channel) and >254 (saturated pixels). The low (background/autofluorescence) threshold was determined by measuring the average pixel value of a section of the image that contained a faint cytoplasmic signal (assumed to be autofluorescence) but no fibrillar structure (assumed to be SHG from collagen). The low threshold was relatively consistent for each image, with a mean of 3.82±0.25 (SEM), a minimum of 1, and a maximum of 6. The overall average threshold (4) was applied to every image. Saturated pixels occurred occasionally generally due to contamination/dust on the slide, but this incidence was rare. After applying the thresholds, the mean SHG intensity of each image was calculated.

To simulate line-scanning, an algorithm was applied that selected random columns from the two-dimensional images. To simulate point scanning, a random pixel sampling algorithm was applied.

For the lowest sample size of 10, an array of 10 random numbers with values between 1 and the number of columns in the image was created with the “randi” function in MATLAB (line scanning), or 10 random numbers with values between 1 and the number of suprathreshold pixels in the image was generated (point scanning). These arrays contained the indices of the selected lines or pixels for analysis. The mean SHG intensity of the randomly selected lines or pixels in each image was then calculated. This process was repeated logarithmically increasing sample sizes, including 10, 100, 1000, and 10,000 lines or pixels. This process was repeated ten times to estimate variation in sampling. Figure 1 shows an example, with the original image, the thresholded image, and simulated point and line sampling. The images are false-color mapped (rainbow, black, and blue representing low grayscale intensity, red representing high).

## 3 Results

### 3.1 Average Second Harmonic Generation Signal Shows Significant Differences Between Tissue Types

**Fig. 2:**
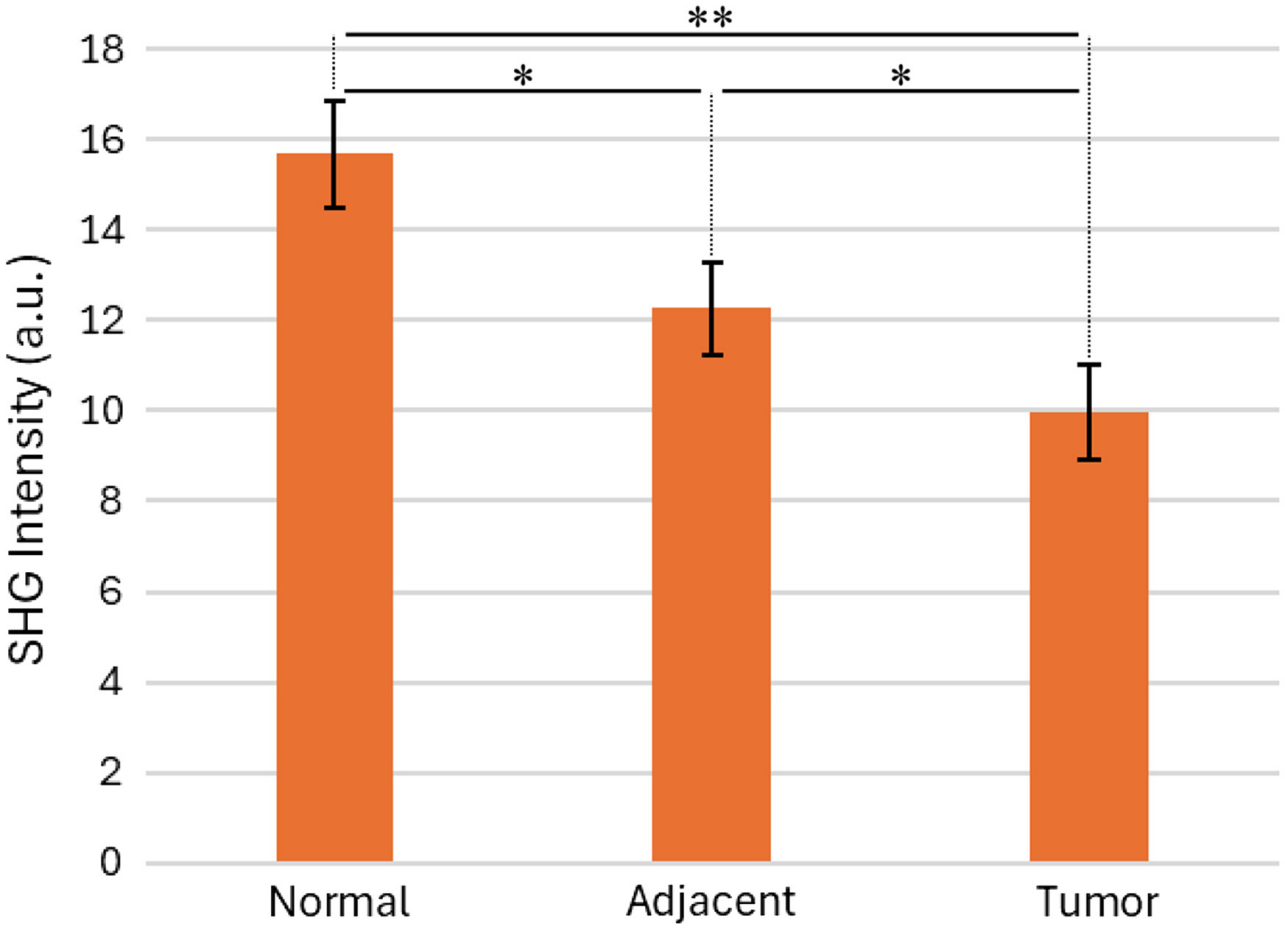
Mean SHG pixel value in images of normal, tumor-adjacent, and tumor tissue. Using a paired t-test, p-value<0.05=*, p-value<0.01=**.

Figure 2 shows that the overall mean grayscale value of SHG images from each tissue type (normal, tumor-adjacent, and tumor) was significantly different from each other tissue type. The significance levels shown in Fig. 2 are based on a paired t-test; paired and unpaired t-test results are summarized in Table 2.

**Table 2:** Summary of paired and unpaired significance, with p<0.05=* and p<0.01=**.

		Adjacent/tumor	Normal/adjacent	Normal/tumor
Paired	p-value	0.014*	0.035*	0.008**
Unpaired	p-value	0.155	0.050*	0.003**

Images of normal tissue had the highest mean SHG image grayscale value, followed by tumor-adjacent and tumor tissue.

The significance levels shown in Table 2 suggest that the average grayscale value of SHG images is different in mucosal collagen images obtained in the three different tissue types, with an emphasis on the high level of significance between normal and tumor tissue (p<0.01 for both paired and unpaired t-tests).

### 3.2 Qualitative Image Comparison Matches Quantitative Trends of Second Harmonic Generation Intensity

**Fig. 3:**
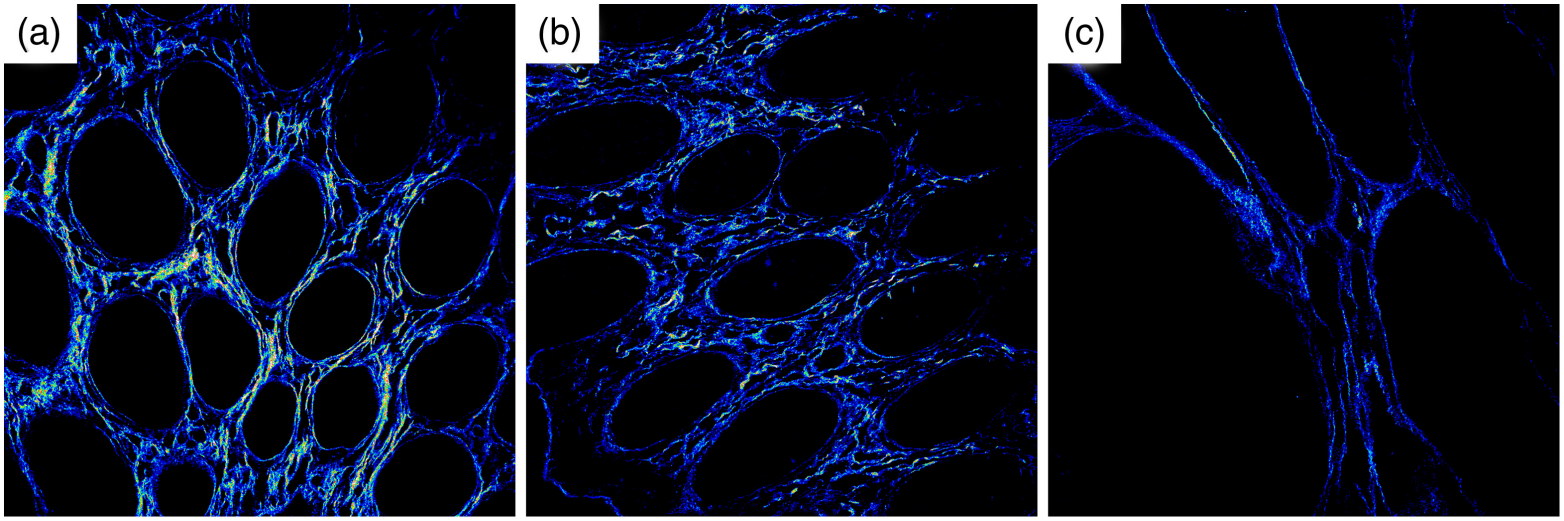
SHG images of normal (a), tumor-adjacent (b), and tumor (c) mucosal structures in the same subject. These images have been thresholded and have the same brightness and contrast settings to allow for a true intensity comparison.

Figure 3 is an example of normal, tumor-adjacent, and tumor tissue. These images show the overall decreasing grayscale value trend of the quantitative data shown in Fig. 2. Note that, in addition to the lower density of the collagen structures in the tumor, there is also an average decreasing intensity of the signal from collagen fibers progressing from the normal tissue to tumor tissue.

### 3.3 Random Point and Random Line Sampling Shows Significance for Differentiating Tissue Types

**Fig. 4:**
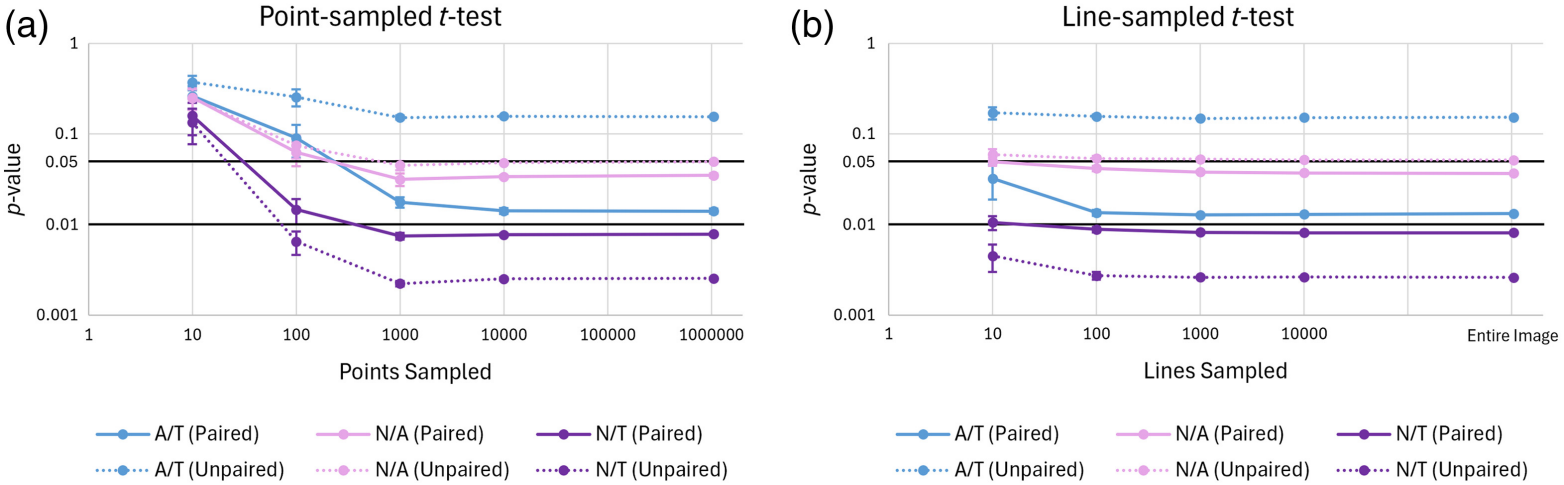
p-value improves as more (a) points and (b) lines are sampled in the thresholded image for comparisons between adjacent/tumor (A/T), normal/adjacent (N/A), and normal/tumor (N/T) groups, for both paired (solid line) and unpaired (dashed line) t-tests. (a) Significance levels of p<0.05 are achieved for groups N/A (paired and unpaired) and A/T (paired) for sampling levels above 1000 points. Significance levels of p<0.01 are achieved for group N/T (paired and unpaired) for sampling levels above 1000 points. (b) Significance levels of p<0.05 are achieved for groups N/A (paired and unpaired) and A/T (paired) for sampling levels above 100 lines. The significance level of p<0.01 is achieved for group N/T (paired and unpaired) above 100 lines.

Analysis of the randomly sampled images reveals that the paired p-value for comparison of grayscale value among all three tissue types shows significance and generally resembles the entire image, after sampling only 1000 pixels [Fig. 4(a)] or 100 lines [Fig. 4(b)]. As expected, line sampling performs better than point sampling for 10 and 100 lines/points, because there were tens to hundreds of analyzable points per line. These results suggest that non-imaging, randomly sampled SHG intensity measurements are likely to be sufficient for differentiating between normal, tumor, and tumor-adjacent tissue in the colon.

## 4 Discussion

These results suggest that diagnostic information about colon cancer can be found from randomly sampled SHG intensity measurements in histological sections. Upon analysis of point-sampled SHG images, only 1000 pixels are required to show a significant difference between tissue types. The p-value for line-sampled SHG images is lower than that of point sampling at low numbers of lines/points, achieving significance at 100 lines and optimal performance at 1000 lines. Because random sampling of 1000 points can be implemented quickly and simply with physician and/or patient motion, the slight improvement with line sampling is not likely worth the additional complexity.

We corroborate that the carcinogenesis process affects both the quantity and structural arrangement of collagen fibers, and therefore the intensity of the second harmonic signal that is detected. A comprehensive study showed that the grayscale intensity of SHG images of osteosarcoma, melanoma, and breast cancer is lower in tumors than in normal tissue, which is in agreement with this study on colon cancer.^9^ Limitations of this comparison include a difference in cancer type and a focus on possible endoscopic applications, and therefore mucosal/luminal regions of interest, in this study. A different study found the opposite trend,^10^ in which the intensity of SHG images of tumors was higher than in normal tissue. A possible explanation for the difference is that this study focused on imaging colonic mucosa, whereas the other study imaged deeper colonic structures. This finding could also be due to the desmoplastic response, which can result in higher amounts of collagen in some types of tumors.

In this study, the “adjacent” slides were taken from a region that a surgeon determined had a normal appearance, yet the SHG intensity in this study was significantly lower than normal. This finding supports the presence of a field effect that influences structural changes in the collagen surrounding a tumor, even when changes are not apparent from gross examination. This field effect has also been noted in studies using enhanced backscattering.^14^

Some limitations of the study should be noted. The microscope used in this study uses linearly polarized light to illuminate the sample. The use of linearly rather than circularly polarized illumination could affect the magnitude of the signal obtained because SHG intensity is dependent upon collagen fiber orientation relative to incident light polarization. We ensured our samples were randomly oriented to average out polarization effects.

**Fig. 5:**
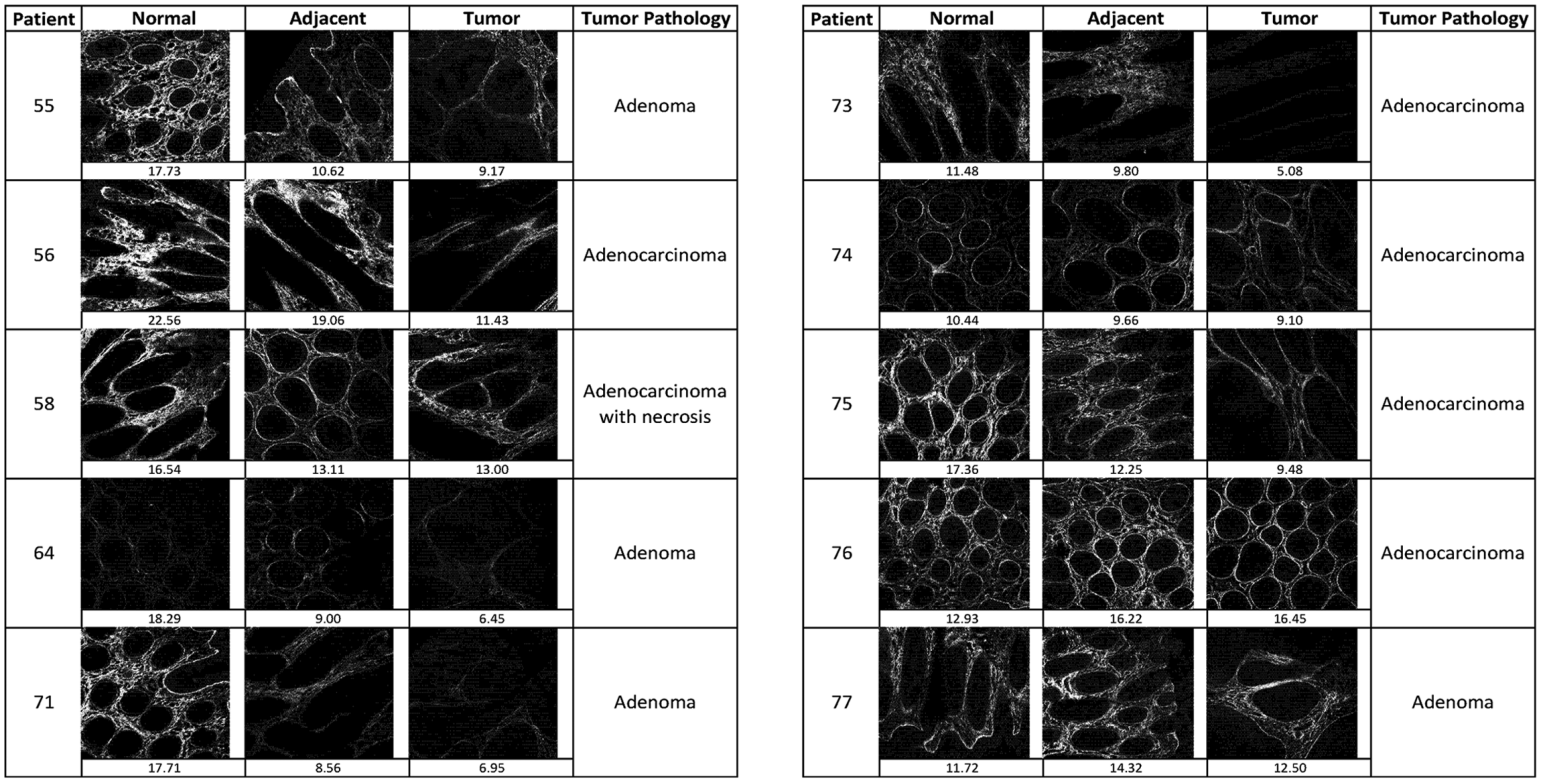
SHG images (.jpg compression) for each subject in this study at normal, tumor-adjacent, and tumor locations, with the mean grayscale value listed below each image. Tumor pathology represents the pathologist’s evaluation of each tumor block’s histological slide. Normal and tumor-adjacent slides were found to be benign colonic mucosa.

Multiple tumor subtypes were included in this study. Although these preliminary results suggest a general trend of decreasing intensity of SHG images with the disease, a larger study that takes tumor subtype into account is justified to investigate subtype-specific trends. Another source of variation in our study is tumor heterogeneity and the small portion of the histological sample that was imaged. Figure 5 shows image thumbnails and descriptions of each subject’s pathology.

Although intensity measurements appear to provide diagnostic information about the presence of tumors, the exclusive use of intensity presents limitations. For example, the intensity measurements in these thin samples could be affected by inconsistency in the thickness of the samples. Also, this study does not consider the effect of multiple scattering such as will occur from thick tissue *in vivo*. Finally, both the collagen organization and concentration contribute to the SHG intensity, so these effects cannot be separated.

### 4.1 Future Directions

Overall, our study suggests that two-dimensional scanning to form an SHG image of collagen may not be necessary for diagnosing colorectal cancer. If these results are also found *in vivo* in intact tissue, there may be potential for the development of a simplified non-imaging SHG system. Removing the need for scanning with an endoscope greatly simplifies design and reduces cost. This study is the first step toward building a non-imaging endoscope that may provide additional information and help improve diagnostic accuracy during or after a colonoscopy.

## 5 Conclusion

With a high incomplete resection rate for colorectal tumors, colonoscopy technology needs improvement to provide accurate tumor margin assessment. SHG measurements, while widely known to be effective in comparing cancerous tissue to normal tissue, have been limited primarily to *ex vivo* applications in part due to the complexity and expense of miniaturized scanning mechanisms. This study on colon mucosa suggests that two-dimensional scanning to obtain an image may not be necessary to obtain enough data on a mass to predict whether it is a tumor. SHG intensity alone, averaged over lines of pixels or random pixels in an image, may contain enough information to differentiate between normal tissue and tumor/tumor-adjacent tissue. This study is the first step toward implementing SHG technology in a simple small endoscope that could be introduced through the working channel of a colonoscope to provide additional diagnostic information.

## Data Availability

All data in support of the findings of this paper are either available within the article or are available in the University of Arizona Research Data Repository.
